# A Child Infected with COVID-19 in China—A Case Report

**DOI:** 10.1093/tropej/fmaa090

**Published:** 2021-08-18

**Authors:** Chunxia Lin, Beilong Zhong, Fangfang Zheng, Xinming Guo, Yi Guo, Zhizhong Deng, Chuanxin Zhou, Yangbin Guo

**Affiliations:** 1 Department of Pediatrics, The Fifth Affiliated Hospital, Sun Yat-sen University, Zhuhai, Guangdong 519000, China; 2 Department of Thoracic Surgery, The Fifth Affiliated Hospital, Sun Yat-sen University, Zhuhai, Guangdong 519000, China; 3 Department of Pharmacy, The Fifth Affiliated Hospital, Sun Yat-sen University, Zhuhai, Guangdong 519000, China

**Keywords:** COVID-19, SARS-CoV-2, children, nucleic acid amplification test, fecal–oral transmission

## Abstract

A 16-month-old boy was admitted with cough for 2 days and fever for 1 day. Chest computed tomography (CT) scan of the child revealed large areas of ground-glass opacities in both lungs. Nucleic acid amplification tests (NAATs) were performed repeatedly to detect severe acute respiratory syndrome coronavirus 2 (SARS-CoV-2), but the results were all negative. On day 13 of hospitalization, no clinical symptoms except diarrhea were present in the patient, and re-examination by chest CT revealed lesion shrinkage, but the NAAT on throat swabs was positive. On day 22 of hospitalization, the NAAT on throat swabs was negative and the fecal samples were positive. Positive fecal samples nucleic acid lasted for 62 days. Suggesting that pediatric patients may be important sources of infection during the recovery phase of clinical symptoms and whether SARS-CoV-2 has fecal–oral transmission needs further study.

Coronavirus disease 2019 (COVID-19) is still a serious global problem. All populations are generally susceptible to COVID-19 [1]. The clinical characteristics of COVID-19 in children are different from those in adults.

## PATIENT PRESENTATION

A 16-month-old boy from Hubei, China, was hospitalized in our hospital on 26 January 2020. Two days before, the patient had cough, mainly dry, no shortness of breath, no cyanosis and no fever. After treatment with cough medicine at a local clinic for 2 days, the coughing had worsened. He developed a fever (38.3°C) and began to have a runny nose. The patient was from an epidemic area and had a history of close contact with COVID-19 patients. He was admitted to a negative-pressure isolation room in the Department of Infectious Diseases of our hospital. Physical examination results at admission were as follows: body temperature 38.3°C, heart rate 116 beats/min, homogeneous heart rhythm, respiratory rate 25/min, blood pressure 92/56 mmHg, percutaneous arterial oxygen saturation ∼0.98, clear-minded and energetic, smooth breathing, no cyanotic lips, symmetrical respiratory motion of the bilateral hemidiaphragms and soft abdomen with no tenderness or rebound tenderness. The neurological examination was normal. Laboratory examination results after admission were shown in Table 1. The result of the nucleic acid amplification tests (NAATs) for severe acute respiratory syndrome coronavirus 2 (SARS-CoV-2) was shown in Table 2 (A and B).

**Table 1:** Infection indicators, liver and kidney function, coagulation function and heart enzyme levels in the child with COVID-19

	WBC (×10^9^/l)	NEU (×10^9^/l)	LY (×10^9^/l)	MONO (×10^9^/l)	CRP (mg/l)	PCT (ng/ml)	ALT (U/l)	ALB (g/l)	UREA (mmol/l)	CREA (μmol/l)	FIB (g/l)	D-Dimer (ng/ml)	CK-MB (U/l)
Day 1	9.17	3.11	4.96	0.97	9.40	<0.10	15.00	39.50	2.5	17.00	4.19	92.00	25.80
Day 5	6.10	2.19	3.19	0.54	0.81	0.20	18.90	37.80	2.4	20.70	2.63	126.00	40.80
Day 15	5.98	1.66	3.69	0.44	0.31	0.11	16.50	40.50	3.80	24.40	2.42	157.00	38.90
Day 34	6.16	1.65	3.92	0.47	–	<0.1	19.20	43.60	4.30	21.70	2.03	242.00	42.00

Notes: WBC is the white blood cell count, and the reference range is 3.5–9.5 × 10^9^/l. NEU is the neutrophil count, and the reference range is 1.8–6.3 × 10^9^/l. LY is lymphocyte count, and the reference range is 1.1–3.2 × 10^9^/l. MONO is the monocyte count, and the reference range is 0.1–0.6 × 10^9^/l. CRP is C-reactive protein, and the reference range is 0.068–8.2 mg/l. PCT is procalcitonin, and the reference range is 0–0.5 ng/ml. ALT is alanine aminotransferase, and the reference range is 9–50 U/l. ALB is albumin, and the reference range is 40–55 g/l. The reference range of urea is 2.0–8.2 mmol/l. CREA is creatinine, and the reference range is 57–111 μmol/l. CK-MB is creatine kinase–myocardial band, and the reference range is 0–25 U/l. FIB is fibrinogen, and the reference range is 2.38–4.98 g/l. The reference range of D-dimer concentration is 0–243 ng/ml.

**Table 2:** Result of NAAT for detection of SARS-CoV-2

A	Day 1	Day 2	Day 3	Day 4	Day 6	Day 8	Day 10	Day 13	Day 14	Day 15	Day 17	Day 19	Day 21	Day 22	Day 23	Day 24	Day 26	Day 27	Day 28	Day 29
Throat swabs	−	−	−	−			−	+	+	+	+	−	+	−	−	±	−	−	−	−
Fecal samples					−	−			−	−			−	+	+	+	+	+	+	

B	Day 31	Day 32	Day 34	Day 36	Day 39	Day 41	Day 43	Day 44	Day 45	Day 47	Day 49	Day 50	Day 62	Day 71	Day 74	Day 77	Day 79	Day 80	Day 84	Day 90

Throat swabs		−													−					−
Fecal samples	+	+	+	+	+	+	−	+	+	+	−	−	+	−	+	+	−	+	−	−

Note: − represent negative; + represent positive.

**Fig. 1:**
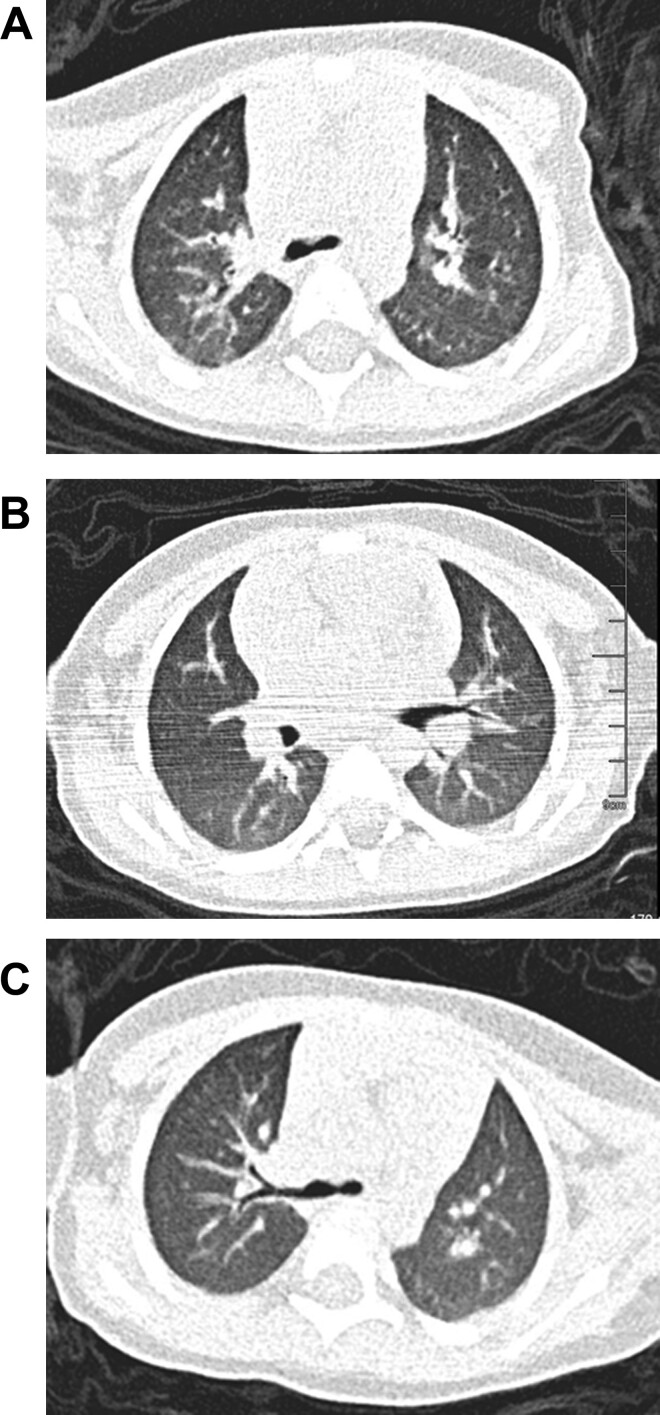
Chest CT images of the child with COVID-19. (**A**) Multiple inflammations in both lungs; large areas of GGOs in both lungs, possibly caused by poor breath-hold performance (day 1 of hospitalization). (**B**) Less inflammation in the right upper lobe than that on day 1 (day 8 of hospitalization). (**C**) Chest CT images displayed normal lungs (day 17 of hospitalization).

Imaging examination: Chest computed tomography (CT) images taken on day 1 of hospitalization showed multiple inflammations in both lungs. (Fig. 1A). Chest CT images taken on day 8 of hospitalization revealed less inflammation in the right upper lobe than that on day 1 (Fig. 1B). Chest CT images taken on day 17 of hospitalization displayed normal lungs (Fig. 1C).

On day 1 of hospitalization, combined with his epidemiological history and his family members were diagnosed with COVID-19, NAAT on throat swabs for SARS-CoV-2 were run regularly. The child presented with respiratory symptoms and typical pulmonary CT changes, refer to ‘Diagnosis and treatment protocol for COVID-19 (fourth trial version)’ [2] and ‘Recommendations for the diagnosis, prevention and control of the 2019 novel coronavirus infection in children (first interim edition)’ [3]. The child was given an intravenous infusion of immunoglobulin (400 mg/kg) for 5 days, received antivirus nasal spray of recombinant human interferon α-2b for 2 weeks. Repeated NAATs for SARS-CoV-2 were necessary. On day 2 of hospitalization, the patient had no fever but developed diarrhea with loose stools two to three times a day. We started to detect NAAT on fecal samples, but the result was negative. The cough subsided on day 9 of hospitalization. On day 13 of hospitalization the NAAT on throat swabs was positive, so the patient was diagnosed with COVID-19. On day 17 of hospitalization, the chest CT images revealed normal lungs. On day 22 of hospitalization, the NAAT on throat swabs was negative, but fecal samples were positive. The child still had diarrhea and continued to be isolated. One week after gastrointestinal symptoms disappear, the NAATs on fecal samples was negative on 18 April (last 62 days), and the patient was discharged on 25 April.

## DISCUSSION

The symptoms of children confirmed with COVID-19 are mostly mild [3]. The manifestations of the patient in this study were respiratory and gastrointestinal symptoms with mild clinical symptoms [4–6]. Early on, though, his NAATs were negative, but he had a history of close contact with the diagnosed patient, and his chest CT examination was consistent with changes in SARS-CoV-2 infection. Before the NAAT was positive for SARS-CoV-2, the child had initial positive chest CT consistent with COVID-19, which suggest Chest CT had high sensitivity for diagnosis of COVID-19 [7].

After the respiratory symptoms completely disappeared, the NAAT on throat swabs was positive for SARS-CoV-2 (on day 13 of hospitalization), which suggests a longer incubation period for SARS-CoV-2 infection in children [8]. Two days after the chest CT images revealed normal lungs (On day 17 of hospitalization), the NAAT on throat swab was negative. Between days 17 and 24, there are a mix of positive or negative results for the throat swab, it maybe has associated with the reduced viral load or the sample collection operations. This is consistent with the conclusion of Chen *et al*. that, although children exhibit mild infection, there is still continuous excretion of SARS-CoV-2 after clinical recovery [4, 5]. Asymptomatic infection or excretion of SARS-CoV-2 in the latent period and recovery period seem to be important sources of infection. Therefore, COVID-19 with mild symptoms or asymptomatic infection must be managed to stop them from becoming transmission sources of community infection.

The viral RNA and viral nucleocapsid protein were examined in gastrointestinal tissues from one case of severe COVID-19 in our hospital [9]. In this study, the child had gastrointestinal symptoms and NAAT on fecal samples was positive for SARS-CoV-2. It provided evidence for gastrointestinal infection. The child continued to have positive results in stool after showing negative results in respiratory samples, indicating that potential fecal–oral transmission can last even after viral clearance in the respiratory tract [9]. In a case report in Hainan, China, SARS-CoV-2 was detected in the fecal samples of a pediatric patient and in the fecal samples of the mother, who had asymptomatic infection by NAAT [10]. Moreover, the team of our hospital and others have reported that SARS-CoV-2 RNA was detected in feces from patients infected with SARS-CoV-2 [9, 11, 12]. Fecal–oral transmission could be an additional route for viral spread, we should take some action to control the spread of the virus from fecal–oral transmission. Therefore, whether SARS-CoV-2 can spread by fecal–oral transmission needs to be further studied through animal experiments. 
